# Using Principal Components Analysis to Visualize Motion and Mitigate Artifacts in Dynamic Optical Coherence Tomography

**DOI:** 10.1002/jbio.70315

**Published:** 2026-06-23

**Authors:** Alejandro Martínez Jiménez, Adrian Bradu

**Affiliations:** ^1^ Applied Optics Group, School of Engineering, Mathematics and Physics University of Kent Canterbury UK; ^2^ Departamento de Física, Facultad de Ciencias Ambientales y Bioquimica Universidad de Castilla‐La Mancha, Campus de la Fábrica de Armas Toledo España; ^3^ Instituto de Nanociencia, Nanotecnología y Materiales Moleculares (INAMOL), Universidad de Castilla‐La Mancha, Campus de la Fábrica de Armas Toledo España

**Keywords:** dynamic OCT, histopathology, optical coherence tomography, principal component analysis

## Abstract

Dynamic Optical Coherence Tomography (DOCT) is an advanced imaging technique that uses temporal fluctuations in OCT signals to improve contrast and enhance visualization of dynamic processes such as motion and metabolic activity. Although various methods for implementing the DOCT algorithm have been proposed, the use of Principal Component Analysis (PCA), a commonly used technique in medical imaging, remains relatively underexplored in this area. Our study demonstrates that selecting only the most significant principal components in PCA can substantially reduce artifacts from strong specular reflections, particularly when high‐numerical‐aperture microscope objectives are used. Furthermore, by using a small number of principal components, we can isolate movement within the sample, successfully reconstruct volumetric images, and create thin, histology‐like sections of bovine kidney tissue, avoiding the need for complex, time‐consuming techniques used in clinical histopathology.

## Graphical abstract

We demonstrate that Principal Component Analysis (PCA) can be used in dynamic OCT to substantially reduce artifacts from strong specular reflections, isolate movement within the sample, and create thin, histology‐like images, thereby avoiding the need for complex, time‐consuming techniques used in clinical histopathology.

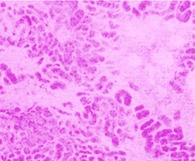

## 1 Introduction

Principal Component Analysis is a technique that has proven useful in medical imaging. It operates on the idea that signals of interest and artifacts lie in separate, orthogonal subspaces within the data. By converting correlated variables into uncorrelated principal components (PCs), PCA helps separate signal energy based on variance. High‐variance components usually represent dominant, stable structural features, while lower‐variance components may reflect dynamic physiological signals or noise. PCA's flexibility has enabled its successful application across diverse areas, including ultrasound angiography [1], magnetic resonance imaging [2, 3], and X‐ray computed tomography (CT) [4, 5].

Since its introduction in the early 1990s, optical coherence tomography has evolved from a structural imaging tool into a method for assessing the physiological state of biological tissue [6]. By taking repeated measurements at fixed locations using protocols such as the MB‐scan protocol [7], OCT can detect motion at scales ranging from blood flow in retinal capillaries to organelle transport within living cells [8, 9]. Although many OCT applications involve repetitive data acquisition and are therefore amenable to PCA, relatively few OCT studies have used PCA to date.

The first example of using PCA is DOCT, in which an MB‐scan protocol is used to visualize intracellular motion and individual cells, conventionally by applying a Fourier Transform (FT) to the data along the time axis [10, 11, 12]. The resulting spectra are integrated over three frequency ranges, each up to several Hz, corresponding to channels in the RGB color space. Very low frequencies, usually below 0.1 Hz, are attributed to static tissue. However, the FT‐based technique requires collecting many B‐scans over long periods, which may be impractical. To address this, a method using the HSV color space has been proposed as an alternative [13, 14]. In this approach, hue values represent the speeds of moving scatterers, calculated from temporal phase drifts, and the value components are derived from all principal components obtained from PCA of the complex data. This method produces motion maps comparable to those generated by the FT‐based approach but needs significantly fewer B‐scans per position, reducing imaging time to as little as 40 ms [13], compared to the several seconds typical of FT‐based techniques.

A second example of PCA usage is in optical microangiography (OMAG), which also relies on complex data and has been developed to distinguish static tissue from capillary blood flow, responsible for fluctuations at several 100 Hz [15]. OMAG usually uses a reference arm or lateral‐scanning modulation to separate static and dynamic signals with closely spaced frequencies. Recently, researchers have implemented OMAG digitally, removing the need for hardware modulation by using a variant of PCA called robust PCA (RPCA) [16]. However, traditional PCA methods with singular value decomposition (SVD) and eigenvalue decomposition are unreliable in OMAG because they often introduce motion artifacts when sampling high‐frequency signals with few B‐scan repetitions. A modified RPCA algorithm, known as frequency‐constrained RPCA, has been successfully used to segment dynamic features in OCT, achieving good accuracy with 1350 B‐scans per location [17].

Finally, recently, a high‐performance, open‐source platform for real‐time digital hologram rendering and analysis that uses PCA to filter noise and extract specific features in holographic OCT has been demonstrated [18, 19].

To our knowledge, aside from the reports mentioned, no other studies on the use of PCA to sense motion in OCT have been published.

Resolving subcellular structures, often smaller than a micron, requires DOCT instruments with high transverse resolution, typically achieved with high‐numerical‐aperture (NA) objectives. This creates a significant trade‐off: improved transverse resolution results in a substantial reduction in depth‐of‐focus (DOF). Signal degradation in out‐of‐focus regions particularly harms DOCT. In these areas, signals from a single moving scatterer are dispersed and averaged with signals from blurred surroundings, which may be static or moving in different directions. This spatial integration results in two main effects: damping of the dynamic signal, due to mixing low‐variance dynamic signals with high‐variance static background, and a decrease in signal‐to‐noise ratio (SNR). Several studies have identified errors in measuring movements due to out‐of‐focus regions. For example, in reference [20], theoretical and simulation evidence show that imaging outside the focal plane can misinterpret non‐uniform axial motion as lateral movement, whereas Wei et al. [21] show that defocus weakens OCT angiography (OCTA) image quality more than it does in structural OCT, since fine capillaries may disappear from the angiogram and larger vessels may appear artificially thickened. To address the need to increase DOF in DOCT, [13] describes a hardware solution using an optical fiber tip equipped with a mirror‐tunnel‐based depth‐of‐focus extension.

In this paper, we demonstrate that using the principal components of complex data generated by conventional PCA enables reliable visualization of motion in DOCT, even with a low number of B‐scans per location, without relying on FTs or temporal phase drifts to infer motion, as demonstrated in previous studies. To eliminate the effects of out‐of‐focus regions, a Gabor‐domain (GD) fusion method [22, 23, 24] was implemented in a phase‐sensitive dynamic OCT instrument. Multiple datasets captured at different focal planes were computationally fused to combine information from each, resulting in a single, fully focused volumetric image. Additionally, the use of PCA reduced artifacts, including saturation artifacts caused by specular reflections, which are common in Gabor‐fusion systems, and proved useful for creating histology‐like images of bovine kidney tissue without the need for complex, time‐consuming procedures used in clinical histopatology, such as dehydration, infiltration, sectioning, or staining.

## 2 Methods and Methodology

### 2.1 Experimental Setup

**FIGURE 1:**
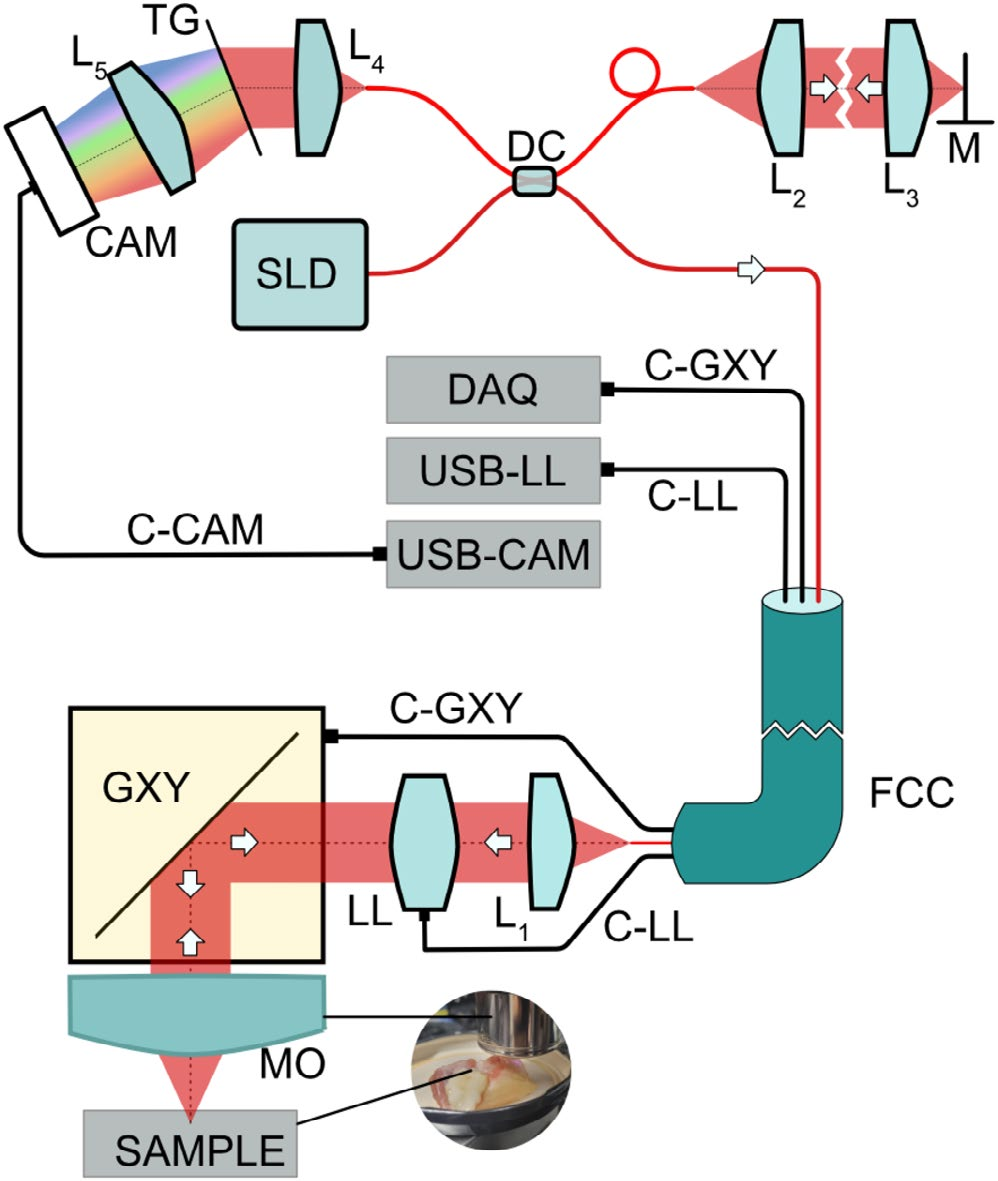
Schematic diagram of the OCT instrument. L_1_–L_5_: Achromatic lenses; MO: Microscope objective; LL: Liquid lens; FCC: Flexible cable conduit; C‐LL, C‐GXY, and C‐CAM: Electric cables connecting the liquid lens and the camera to the USB ports of the computer and the galvos to the multi‐function I/O DAQ, respectively. USB‐LL and USB‐CAM are the USB driver boards of the liquid lens and camera, respectively; TG: Transmission diffraction grating; GXY: A pair of orthogonal galvo‐scanners; DC: Directional coupler; SLD: Superluminescent diode; CAM: Linear camera. In the inset, a picture of the microscope objective and of a sample under investigation is presented.

In Figure 1, a schematic diagram of the developed OCT instrument is presented. Light from a superluminescent diode (SLD) with a central wavelength of 840 nm and a spectral bandwidth of 45 nm was employed. Light from the SLD is directed toward the sample via a 50/50 directional coupler DC. In the sample arm, light passes through an achromatic lens (L_1_), a variable‐focus liquid lens (LL, diameter 5.8 mm, NIR Coated, A‐58N1‐P20 Varioptic), which enables rapid focus adjustment within the sample, and a microscope objective (MO, Thorlabs, LSM02‐BB). Two orthogonal galvo‐scanners (GXY) scan the optical beam across the sample. Light backscattered by the sample is then recombined with light from the interferometer's reference arm and detected by an in‐house‐designed spectrometer. The spectrometer includes a transmission diffraction grating from Wasatch Photonics with 1200 lpm, which diffracts the optical beam over the pixels of a fast linear camera (Teledyne e2v, model Octoplus USB). Positioning of the galvanometers and triggering of the line cameras is achieved via a multifunction I/O device (National Instruments, model PCIe‐6321) and custom LabVIEW software.

Because the camera acquisition rate was 100 kHz and we aimed for 400 horizontal pixels in the B‐scan images, the fast galvo‐scanner was driven at 125 Hz; therefore, the interscan time between B‐scans was 8 ms. For each pixel of lateral coordinate *x, y*, and axial coordinate *z*, the complex reflectivity was computed using the Master–Slave (MS) approach [25, 26],

(1) $$\begin{array}{c} C_{x,y,z,l}=\sum_{k=1}^{n_{k}}E_{x,y,l,k}\cdot T_{z,k}^{*}=M_{x,y,z,l}\cdot e^{i\varphi_{x,y,z,l}} \end{array}$$

In Equation (1), to enable high‐speed DOCT imaging, only a small number of datasets were recorded for each y‐position, so *l =* 1…0.16. *E* is the value sampled by the pixel *k* of the camera (out of *n*_*k*_ pixels), when the beam is on the sample at position (*x,y*). T* are the numerical inferred spectra, for each axial position z, calculated prior to data acquisition using [23, 24],

(2) $$T_{z,k}^{*}=\frac{{\mathrm{dg}}_{k}}{\mathrm{dk}}e^{-i\left(g_{k}z+h_{k}\right)}$$

In Equation (2), *g* and *h* are functions that account for the inherent non‐uniform sampling of spectral data in wavenumber space by the spectrometer and the unbalanced dispersion in the interferometer, respectively. *n*_*x*_ is the number of horizontal points per B‐scan (400 in our case), *n*_*y*_ the number of points in the *y*‐direction (160 in our case), and *n*_*z*_ the number of axial points, whose value is selected depending on the axial imaging range of interest, but as a rule of thumb, to ensure a good sampling, we ensured that one axial pixel covers maximum 2 *𝜇m* (the experimental measured value of the axial resolution, in air, was 7 𝜇m). *M* is the magnitude, and *φ* is the phase of the OCT signal at (*x, y, z, l*). Each volumetric MB dataset has a dimension of 400 × 200 × 16 pixels^3^ and is acquired in 128 ms, whereas the entire dataset required to build the volumetric image is acquired in 20.48 s. The data is produced and displayed in real time, that is B‐scans are displayed at 125 Hz.

Experimentally, a DOF of 50 μm has been measured by axially translating a flat mirror through the MO focal plane. To produce a fused Gabor image of maximum axial range of 1.9 mm (measured experimentally), data must be collected from a minimum of *R* = 38 focusing positions [27]. However, when imaging highly scattering samples, fewer acquisitions suffice.

**FIGURE 2:**
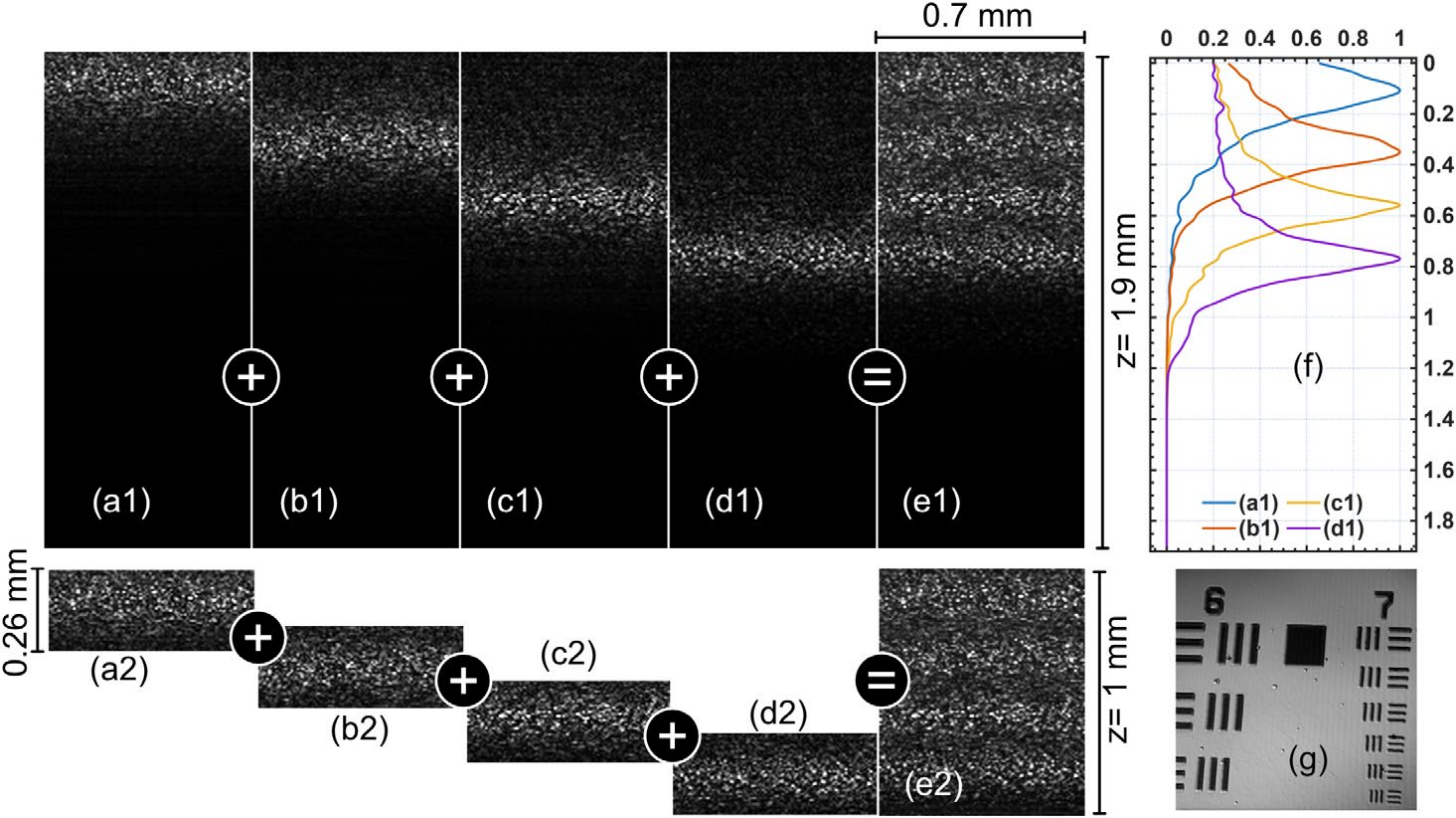
(a1–d1) B‐scan images (xz‐plane) of a titanium dioxide resin with various focusing axial positions; (e1) B‐scan produced by fusing (a1–d1); (f) Normalised profiles obtained by averaging the A‐scans from (a1, b1, c1 and d1); (a2–d2) B‐scans images of the titanium dioxide resin, of narrow axial imaging range; (e2) B‐scan produced by concatenating (a2–d2); (g) *en‐face* OCT image of an USAF test target.

As an illustration, four B‐scan images (*xz*‐plane) of a titanium dioxide resin with various focusing axial positions are shown in Figure 2a1–d1. As one can observe in Figure 2f, where normalized axial reflectivity profiles obtained by averaging A‐scans, the width of the confocal gates is wider than 50 μm. The refractive index of pure titanium dioxide is around 2.5, and that of the resin is around 1.5; therefore, a DOF of around 200 μm measured in air corresponds to around 80–130 μm in the sample. As illustrated in Figure 2e1, a fused Gabor image of the titanium dioxide/resin sample can be produced by fusing around 8 B‐scans collected across the axial range (in Figure 2e1, 4 merged B‐scans cover an axial range of 1 mm).

As a rule of thumb, when imaging biological samples to produce a seamless, artifact‐free B‐scan at the edge of the focusing window, we calculate the number of focusing positions, R, as the ratio of the axial imaging range of interest (AIROI, in μm) to 100 μm. In standard Fourier‐domain OCT, every spectral sweep is processed using a Fourier transform to recover the entire A‐scan. When Gabor fusion is performed across R focal positions, the system must process R full volumes and make use of Gaussian or trapezoidal windows to obtain smooth volumes [24, 28]. When using the MS approach, subvolumes covering only the AIROI can be produced.

The image fusion procedure is illustrated in Figure 2a2–e2, where four B‐scans of AIROI = 0.26 mm are used to generate the fused image illustrated in Figure 2e2 of axial range 1 mm. To produce the fused image, one could simply concatenate the 4 images if their axial ranges were 0.25 mm. However, to avoid errors in determining the precise position of the focusing plane, we acquired images over an axial range of 0.26 mm and used maximum‐intensity projection for the narrow overlapping regions. Figure 2g shows an *en‐face* OCT image of a USAF target. As shown, element 6 of group 7 is clearly resolved at any target position across an axial range of 0.8 mm; therefore, we can assume that the lateral resolution of our instrument, measured in air, was better than 2.19 μm.

The concept of combining multiple focal planes acquired with a tunable lens to extend the depth of field in OCT has been established in the literature, most notably through the Gabor Domain OCM (GD‐OCM) framework introduced by Murali et al. [29] and further developed by Rolland et al. [24]. In those works, the focal plane is sequentially shifted, and the in‐focus regions from each acquisition are fused using a Gabor‐based algorithm, yielding isotropic, high‐resolution imaging across the imaging depth. Variants of this approach have since been demonstrated using deformable mirrors [30], Master–Slave OCT architectures [23], and FPGA‐accelerated processing [31].

Our approach shares the principle of multi‐focus acquisition and fusion but differs in two key respects. First, we operate at a lower objective NA, which relaxes the confocal gating constraint and yields a longer individual depth of focus per acquisition. This reduces the number of focal steps required and, consequently, the time needed to acquire and compute data. Second, the fusion strategy employed here is tailored to the specific requirements of our dynamic imaging application, where the primary goal is not purely morphological resolution but the separation of dynamical modes via PCA. A summary of key performance metrics for some instruments reported in the literature is presented in Table 1.

**TABLE 1:** Key performances of some instruments that implemented the multi‐focus acquisition.

Ref	Type	Lat. res	Sub‐volumes	Speed	Proc. time
29	OCM	2 μm	Continuous over 2 mm	5 fps	No GF, RT
24	GD	2.8 μm	5	5 fps	No RT, NA
[32]	CM	0.46 μm	Continuous over 0.25 mm	7 fps	RT
[33]	GD	2 μm	6 over 0.6 mm	80 kHz	36 s
[34]	GD	2 μm	10–12 over 1.2 mm	55–80 kHz	2 min
C	GD	2.2 μm	4–8 over 0.8 mm	100 kHz	RT, no GPU

Abbreviations: C, current report; CM, confocal microscopy; GD, gabor domain; GF, gabor fusion; NA, data not available; OCM, optical coherence microscopy; RT, real‐time.

### 2.2 Principal Component Analysis. Theory and Implementation

Let the complex data of an M‐B scan be the two‐dimensional array C, in which the number of columns in *C* is the number of repetitions, *r*, at a given (*y, z*) spatial position, whereas *n*_*x*_ is the number of horizontal pixels in each B‐scan.

(3) $$C=\left(\begin{array}{cccc} C_{11} & C_{12} & \ldots & C_{1r}\\ C_{21} & C_{22} & \ldots & C_{2r}\\ \vdots & \vdots & \ddots & \vdots \\ C_{n_{x}1} & C_{n_{x}2} & \ldots & C_{n_{x}r} \end{array}\right)$$

The covariance between two columns of *C* can be computed using,

(4) $$\begin{array}{c} \mathrm{cov}\left(C_{i},C_{j}\right)=\frac{1}{n_{x}}\sum_{k=1}^{n\_x}\left(C_{\mathrm{ik}}-\left\langle C_{i}\right\rangle \right)\left(C_{\mathrm{jk}}-\left\langle C_{j}\right\rangle \right) \end{array}$$

In the most general case, the covariance of *C* is defined as,

(5) $$\mathrm{cov}\left(C\right)={\left(C-\left\langle C\right\rangle \right)}^{T}\left(C-\left\langle C\right\rangle \right)$$

The superscript *T* in Equation (5) signifies the transpose, and the mean values are calculated along each column of *C*. The elements along the diagonal of the squared matrix *cov(C)* are the variances of the columns of *C*. The off‐diagonal elements of *C* are the covariances between its columns. *cov(C)* is a symmetric matrix that describes the correlations among its columns. A positive covariance indicates that the columns are correlated and that their components tend to increase or decrease together. Negative covariance indicates that the columns are negatively correlated and that their components tend to move in opposite directions. A zero covariance means that the columns are not linearly related to each other. The objective is therefore to identify the maximum possible variance for each column of *cov(C)*, as this represents the static features of the data. Specifically, the goal is to determine a vector *p* with *r* elements such that *var(p)* is maximized. This is equivalent to finding a vector *p* such that $p^{T}\mathrm{Cp}$ is maximized [35], which is equivalent to solving the linear equation,

(6) $$\mathrm{cov}\left(C\right)p=\mathrm{\lambda p}$$

Therefore, what we are looking for is to find the pairs of solutions *λp* such that Equation (6) is satisfied. The eigenvectors *p* are the principal components of the data. The eigenvalues *λ* are the variances of the principal components. A crucial consequence of the symmetric nature of the covariance matrix is the orthogonality of the resultant eigenvectors. This mathematical property yields a great statistical benefit: the principal components are statistically uncorrelated. Because the vectors derived from the eigen‐decomposition are orthogonal, the principal components capture independent sources of variation within the data [36]. This mathematically guaranteed decorrelation is essential for effective dimensionality reduction, ensuring that the information retained by the first principal component (PC_1_) is genuinely distinct from that retained by the second principal component (PC_2_), and so forth. PC_1_ captures the maximum possible variance in the dataset and, as a result, can encapsulate the static signal, if one exists. Each subsequent component (PC_2_, PC_3_, etc.) captures the maximum remaining variance not captured by the previous components and, in principle, can be associated with movements within the sample under investigation. In this paper, we reconstructed the data by producing the principal components using the SVD implementation of the *PCA* MATLAB function.

**FIGURE 3:**
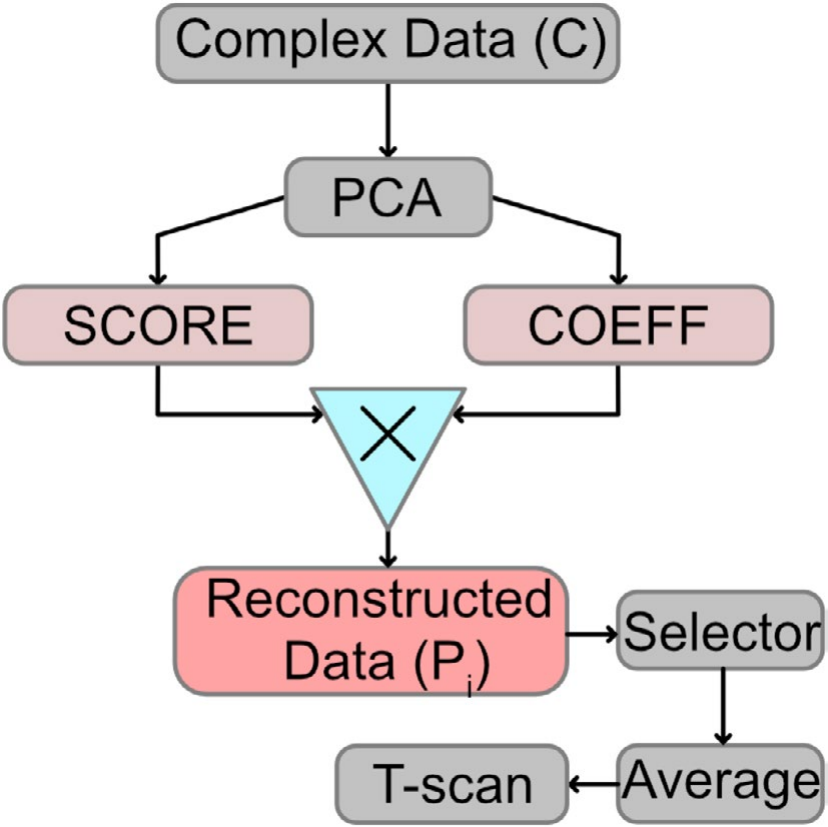
Flowchart illustrating the production of a transverse scan using the PCA.

A flowchart illustrating the production of a transverse scan (T‐scan) using principal components is shown in Figure 3. In our case, the complex data matrix C used to produce it has dimensions 400 × 16. The PCA module returns both the principal component coefficients (COEFF, the eigenvectors of the covariance matrix) and their scores (SCORE, the coordinates of the data points in the new PCA space), which are then multiplied to reconstruct data for each individual principal component “i”. The T‐scan corresponding to a principal component “*i*” is then calculated using,

(7) $$P_{i}=\mathrm{var}\left({\text{SCORE}}_{i}*{\text{COEFF}}_{i}\right)$$

The Selector block chooses only the components required for the final T‐scan, which are then averaged by the “Average” block.

### 2.3 Real‐Time Performance and Computational Cost

The processing pipeline consists of two main stages with distinct computational demands.

The first stage, complex OCT volume reconstruction, benefits directly from the Master–Slave architecture, which selectively delivers data from selected depths during lateral scanning, thereby avoiding the need to process the full axial range at every step. Combined with the Gabor fusion scheme, this makes the reconstruction stage computationally efficient and amenable to parallelization. To ensure real‐time operation, B‐scan images must be reconstructed and displayed on the screen faster than the interscan time between B‐scans. In our case, the time required to process data and display a B‐scan image of size 400 × 200 pixels using our in‐house‐developed LabVIEW software on a computer equipped with a 12‐core Intel Core i9‐12700KF processor was 2.8 ms.

The second stage, PCA computation, involves an eigen decomposition of the data covariance matrix **cov(C)**, whose cost scales as $O\left(N^{3}\right)$ if its size is *N* × *N*. In practice, this can be significantly reduced by retaining only the leading k≪N components via truncated SVD, with cost $O\left(kN^{2}\right).$ Our MATLAB code for computing PCA made use of an NVIDIA GeForce RTX 3060 graphics card. The time required to generate 15 cross‐sectional images of size 400 × 200 corresponding to the 15 principal components, across 5 focusing positions (a total of 75 images), was 304 ms. We must note that this time reflects the current, not fully optimized implementation, and that substantial speed‐ups are achievable through better CUDA implementation or dedicated real‐time processing pipelines.

### 2.4 Sample Preparation

A culture of H358 human lung cells from bronchioalveolar carcinoma (H358; ATCC CRL‐5807), cultured in a Dulbecco's Modified Eagle Medium (DMEM) with glutamine/sodium pyruvate, supplemented with 10% fetal bovine serum and 1% penicillin–streptomycin, and maintained at 37°C with 5% CO_2_, was placed in a Petri dish.

Freshly excited bovine kidney samples were sourced from a local butcher. They were kept at a low temperature to maintain tissue integrity and were imaged within a couple of hours of being brought to the laboratory.

## 3 Results

**FIGURE 4:**
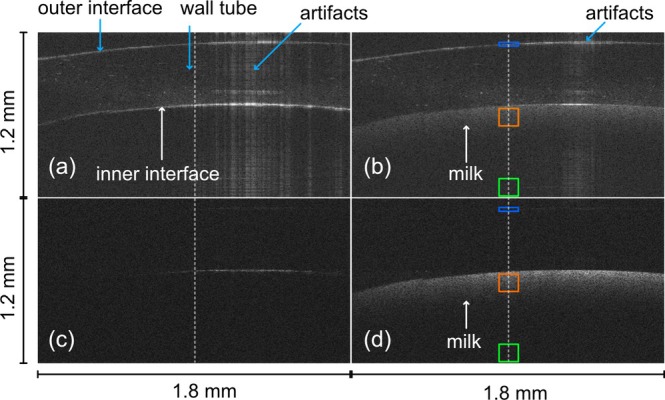
B‐scan images (*xz*‐plane) of a plastic tube. (a, b) show structural images of the tube, without any fluid present inside the tube, and filled with milk, respectively. (c, d) show motion images constructed based on a single principal component. The blue, red, and green rectangles indicated the areas where the data were used to calculate the SNR and CNR (the signal was calculated from the regions of interest indicated by the blue and red rectangles, and the noise from the green rectangle).

To demonstrate that the first principal component, PC_1_, captures the maximum possible variance in the dataset and, as a result, can encapsulate the static signal, we have imaged an initially empty plastic tube. A structural B‐scan image, produced by calculating the magnitude of the complex OCT signal using Equation (1) and orthogonal to the axis of the tube, is depicted in Figure 4a. This image is an average of 16 consecutive B‐scans. The outer and inner separation between the tube and air, as well as various scattering centers, are visible within the wall of the tube. In addition, artifacts are visible due to strong specular reflections (vertical stripes on the right side of the image). After performing PCA and discarding the first principal component, the image shown in Figure 4c was generated. One can observe that:

- The static scatterers within the tube wall are absent from the PC_1_ image.
- The curved lines showing the separation air/outer‐surface and the inner‐surface/air are nearly completely eliminated.
- The vertical stripes due to strong specular reflection are also eliminated.

In Figure 4b, a B‐scan image of the same tube, now filled with milk, is shown. The colloidal particles in the milk suspension are clearly visible below the inner interface. After conducting the PCA analysis, the image in Figure 4d was generated using all principal components except the first. Since higher‐order PCs have lower variance than the first component, the bright pixels in Figure 4d can be linked to the constant Brownian motion of the colloidal particles in the milk suspension.

**FIGURE 5:**
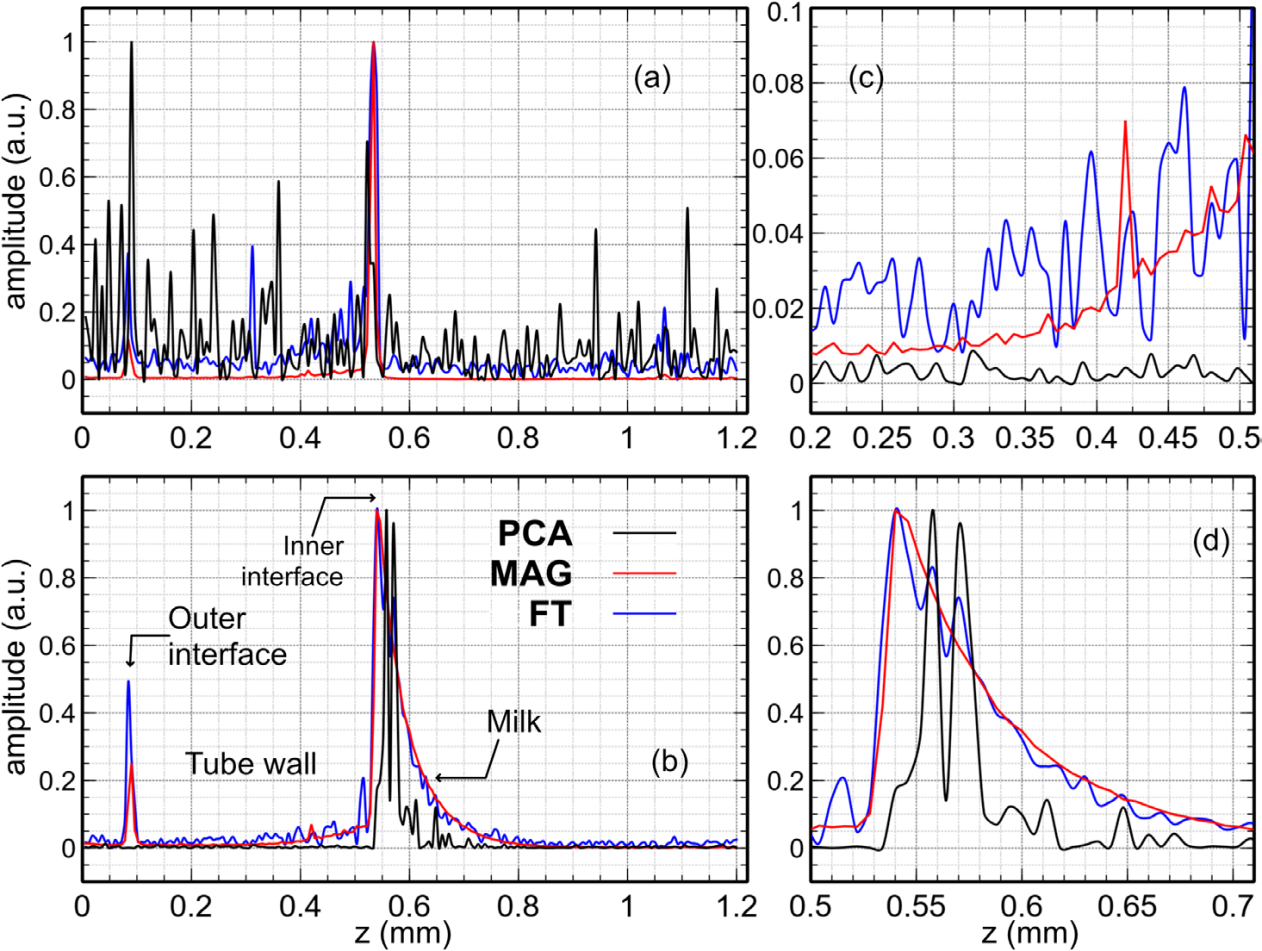
(a) Signal profiles showing structural information (MAG, in red), motion by using the conventional FT‐based approach (FT, in blue), and motion using all principal components but the first (PCA, in black). The plots were generated using data collected at the lateral positions indicated by the dashed lines in Figure 4a,b, respectively. (c, d) are zoomed‐in versions of (a) and (b) respectively.

To better visualize the benefits of using PCA, the normalized signal profiles extracted from the positions marked with dashed lines in Figure 4 are shown in Figure 5. The red plots represent the reflectivity profiles obtained from the structural image, while the black curves are the signal profiles from the images generated via PCA. Additionally, the signal produced by the conventional method for dynamic OCT images [11, 12] is displayed (the FT of the magnitude data along the time axis, in blue). To create this signal, 512 consecutive B‐scans were acquired, and the FFT result was integrated from 0.1 to 62.5 Hz.

In Figure 5a, the red curve clearly indicates the position of the inner and outer interfaces of the tube, while the black and blue curves show rather random fluctuations of the signal along the axial direction. The advantage of using PCA becomes more evident when imaging the milk suspension. The PCA signal exhibits very low values at the air/outer‐surface interface of the tube, a low component due to reflection at the inner‐surface/milk interface, and very small signals from within the walls of the tube (Figure 5b).

When applying the FT approach, the signal within the wall is not mitigated as effectively as with PCA, as shown in Figure 5c. In our case, we eliminated all signals below 0.1 Hz after FT, but this was not enough to prevent perceiving some components within the wall as moving. The PCA method also clearly highlights movement beneath the outer interface. The fluctuations in the signal around *z* = 0.55–0.65 mm are clearly visible using both PCA and FT methods, indicating they are caused by the movement of the colloidal particles.

Another application of PCA can be in experiments on the dynamics of intracellular movement in cells placed in a thin layer of growth medium, where strong specular reflections from the air/growth medium and growth medium/glass interfaces may overlap with the weak scattered light from the cell. Thin layers have a high surface area‐to‐volume ratio. Even slight evaporation can change the height of the growth liquid level during acquisition, which can take seconds to minutes. As a result, the optical path length changes continuously. In phase‐sensitive dynamic OCT, this appears as bulk motion, or “flow,” in the data, masking the true intracellular transport signals. The growth liquid in a wide, open Petri dish is also prone to sloshing or vibrating due to ambient noise. These vibrations induce phase noise that the DOCT algorithms may misinterpret as high metabolic activity. Finally, because the liquid surface is rarely flat, Fizeau fringes can appear due to interference between the front and back surfaces of a thin, wedge‐shaped gap.

**FIGURE 6:**
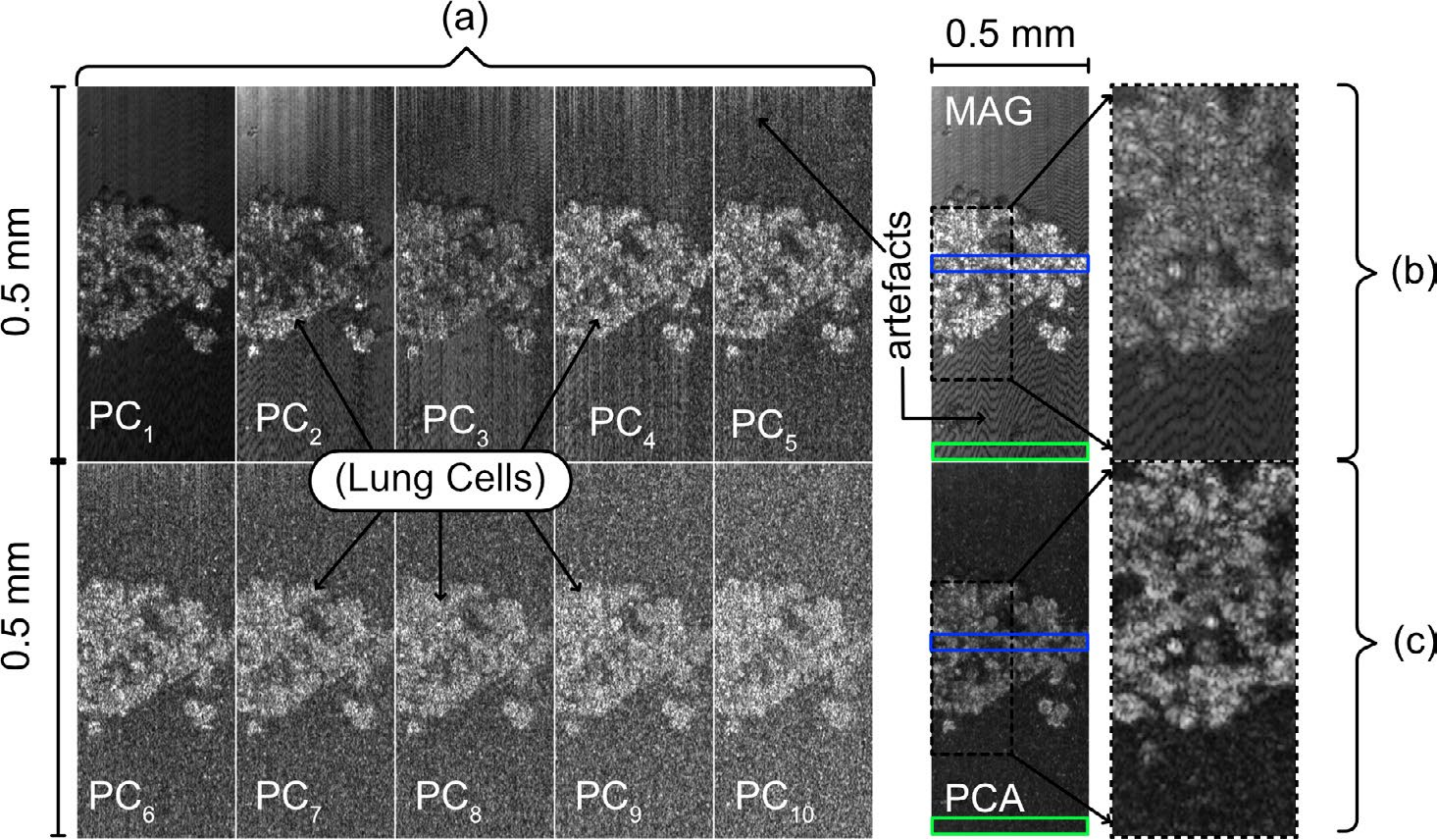
*En‐face* images of a cluster of H583 lung cells. (a) Each image was generated using the first through the 10th principal components. (b) Magnitude image. (c) The PCA image is produced by averaging the images generated using the 6th to the 10th principal components. The zoomed‐in portions of the images, indicated by dashed‐line borders, are a quarter of the full‐size image. The blue and green rectangles indicate the areas where the data were used to calculate the SNR and CNR (the signal and the noise, respectively).

*En‐face* PCA and magnitude images of a thin cluster of H358 culture cells placed on a Petri dish are presented in Figure 6. The images are from an axial position of around 40 μm above the glass.

In Figure 6b, the magnitude image reveals two types of artifacts: Fizeau fringes, with lower density at the bottom and higher density at the top, and fine vertical lines at the top. In Figure 6a, 10 *en‐face* images generated using the first 10 principal components are shown. The first image, created with only PC_1_, should normally display only static or very low‐frequency components. As observed, this image is only slightly affected by the Fizeau fringes and thin vertical lines; however, to detect motion, we need to examine higher‐order PCA components. PC_2_ clearly shows signals with variance caused by the low‐density Fizeau fringes, while PC_3_ shows high‐density Fizeau fringes. All images created using PCA components of order 4 or higher are free of Fizeau fringes but exhibit vertical line artifacts, likely due to specular reflections, with PC_4_ particularly affected. Images generated from PCA orders 6 and higher are artifact‐free; by averaging them, a final artifact‐free motion image is produced that shows better contrast than images from individual PCA components. The image in Figure 6c was generated by averaging images from PCA components 6–10.

PCA does not eliminate artifacts by assuming they occur randomly; rather, it separates contributions by ranking them by their variance. Formally, PCA solves the eigendecomposition of the data covariance matrix $\mathrm{cov}\left(C\right)$, yielding eigenvectors ***p***_***i***_ ordered by their corresponding eigenvalues $\lambda_{1}\geq \lambda_{2}\geq \cdots \geq \lambda_{N}$. Each PC, therefore, captures the mode of variation that explains the most remaining variance, regardless of whether that variation is deterministic or stochastic. Structures sharing similar dynamics, such as tissue layers moving with the same characteristic velocity, contribute coherently to the same eigenvector and are thus grouped within the same PC. The Fizeau fringes, clearly visible in the magnitude image in Figure 6, are still present in the low‐order PCs images, but absent in the higher‐order images. The fact that they are missing from some PCs does not imply that they do not vary over time (in reality, they may vary due to factors such as dehydration of the sample). It simply reflects that their associated eigenvalue $\lambda_{i}$ does not match those associated with specific PCs. If they had missed from PC_1_, for example, it would simply reflect that their associated eigenvalue *λ*_*i*_ is not the largest, that is their spatial pattern does not dominate the covariance structure of the time‐interval OCT volume. As PC_1_ captures the mode of the largest variance, associated with the most stationary or slowly varying signal component, the fringe pattern, having a comparatively smaller $\lambda_{i}$, is naturally relegated to PC_2_ or PC*3*, where, $p_{i}^{T}\mathrm{cov}\left(C\right)p_{i}=\lambda_{i}\ll \lambda_{1}$.

In the present manuscript, the choice of a specific set of PCs that excludes artifact information and includes only movement within the sample was made quite subjectively, and a systematic criterion for PC selection would be desirable. The PCA framework itself provides natural quantitative tools for this purpose, which can potentially be implemented. The ratio $\epsilon_{i}=\lambda_{i}/\sum \lambda_{j}$ itself provides an objective, data‐driven ranking of the principal components. This quantity plays an analogous role to the spectral energy of a frequency band in FFT: just as one would select frequency bands whose energy exceeds a given threshold, PC selection can be formalized as retaining the subset satisfying a cumulative variance criterion, $\sum \epsilon_{i}\geq \tau$, where τ is a threshold parameter. The complementary residual, $1-\sum \epsilon_{i}$ directly quantifies the information loss associated with any given selection, providing a transparent metric for reproducibility. In Fourier‐based methods, the DC component and the narrow band of near‐zero frequencies are routinely discarded for visualization purposes, as they encode the mean background intensity rather than meaningful dynamic information. In PCA, the analogous operation is the exclusion of the components associated with the largest eigenvalues λ_1_,  λ_2_, …, which capture the most stationary or slowly varying structure, the “DC‐equivalent” of the dataset. The robustness of the PC selection is surely governed by the spectral gap between consecutive eigenvalues. A large gap indicates a well‐separated mode that will be stably and reproducibly identified across repeated measurements of the same sample type, whereas a small gap signals overlapping modes that may require additional criteria, such as phase coherence or motion‐style discrimination.

Although the principal components in this study were selected by visual inspection, they show strong correlations with the signal‐to‐noise ratio (SNR) and contrast‐to‐noise ratio (CNR) of the images.

**FIGURE 7:**
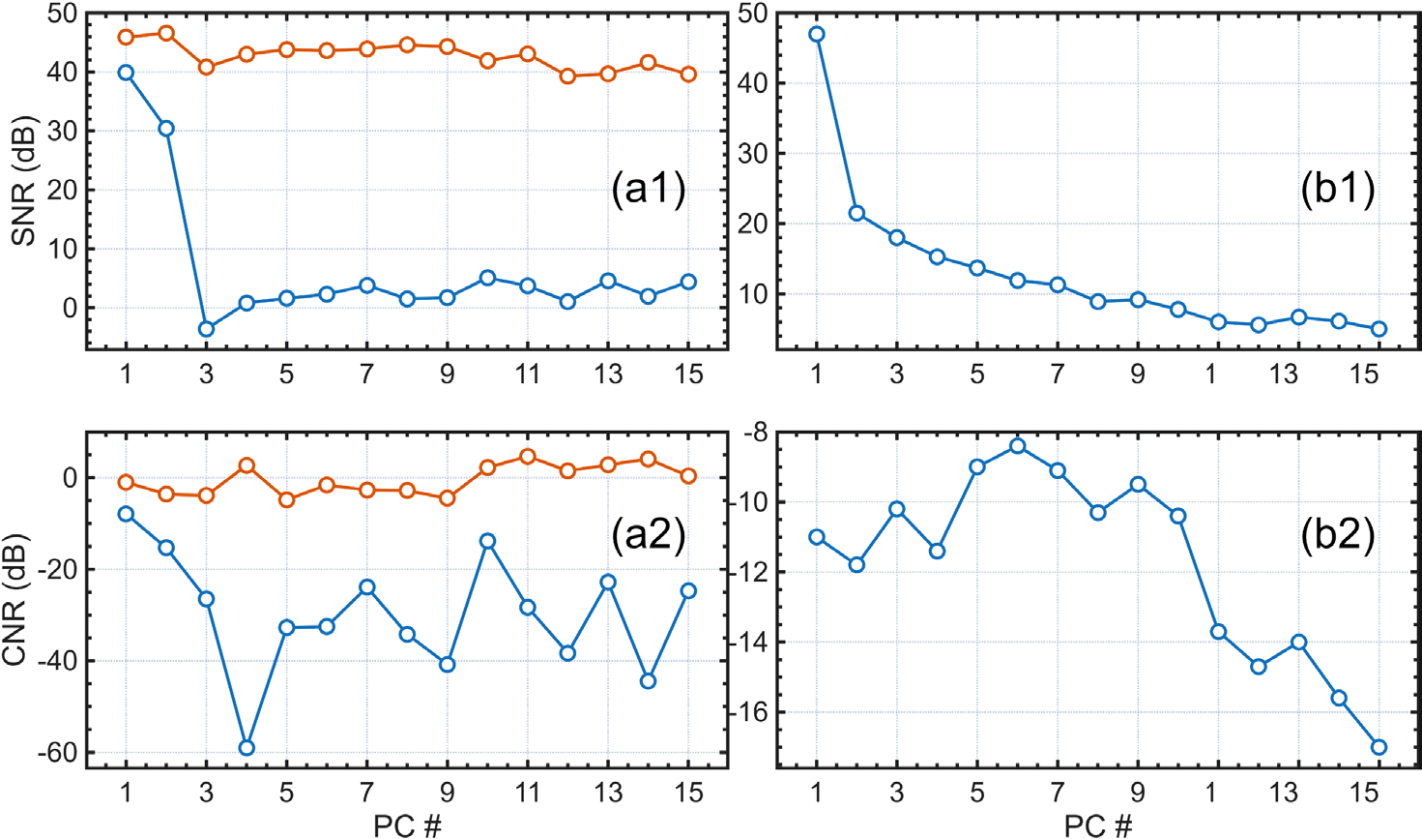
SNR and CNR calculated for the first 15 principal components. (a1, a2) SNR and CNR values at the outer interface of a plastic tube filled with a milk suspension (orange curves) and within the milk suspension. The regions of interest are indicated with blue and orange rectangles in Figure 4. (b1, b2) SNR and CNR values within a cluster of lung cells. The region of interest is indicated with a blue rectangle in Figure 6.

In Figure 7a1,a2, we show the SNR and CNR for the first 15 principal components when the plastic tube filled with the milk suspension was imaged (please see Figure 4). The orange curves correspond to the SNR and CNR values calculated for the regions of interest indicated by orange and blue rectangles in Figure 4. As one can observe, the SNR at the outer tube interface (blue curve in Figure 7a1) is approximately 40 dB higher in PC_1_ and PC_2_ than in the higher‐order PC images, clearly indicating that PC_1_ and PC_2_ must be discarded to image only dynamic events. In the second region of interest (inside the milk suspension), the SNR remains approximately constant across the PCs. The CNR at the outer interface is consistently negative (blue curve in Figure 7a2), which is expected because the features are nearly invisible and the SNR is low. Regardless of the selected PCs, the best CNR at the outer interface is about −8.4 dB for PC_10_‐PC_15_. In the milk suspension, the CNR values are positive for PC_10_‐PC_15_ (orange curve in Figure 7a2), and, as a result, all the PCA images are artifact‐free and show only movement, as illustrated in the (Figure S1). An image obtained by averaging PC_10_‐PC_15_ yields a CNR of 18.1 dB within the suspension, the largest CNR achievable when averaging different sets of PCs.

Using the conventional DOCT technique based on FTs, the SNR and CNR at the outer interface were 35.5 and 4.6 dB, respectively, and within the suspension were 44.7 and 36.8 dB. These results suggest that the PCA method might be more effective at removing signals from stationary particles, while the FT method offers a higher CNR, indicating better overall performance. Nevertheless, these two methods cannot be directly compared because, in the FT approach, an arbitrary 0.1 Hz cutoff frequency was applied, and frequencies up to 62.5 Hz were integrated. Additionally, the PCA method used only 16 repetitions.

Figure 7b1,b2 display the SNR and CNR for the first 15 PCs within the blue rectangles marked on the en‐face images in Figure 6. Since the cell distribution in this area is discontinuous, lower SNR values are observed, especially in higher‐order PCs. All CNR values are negative, indicating noise dominance when CNR < 0 dB. Averaging PCs 6–10 results in a CNR of 2.3 dB and an SNR of 22.3 dB, producing a high‐quality, artifact‐free image labeled as PCA in Figure 6. Analysis across different samples suggests that the optimal set of PCs generally corresponds to the highest CNR, ideally exceeding 0 dB, balancing good SNR with minimal artifacts. This is supported by the analysis we present in the (Figures 2S and 3S), which show data and images produced from imaging bovine kidney tissue.

**FIGURE 8:**
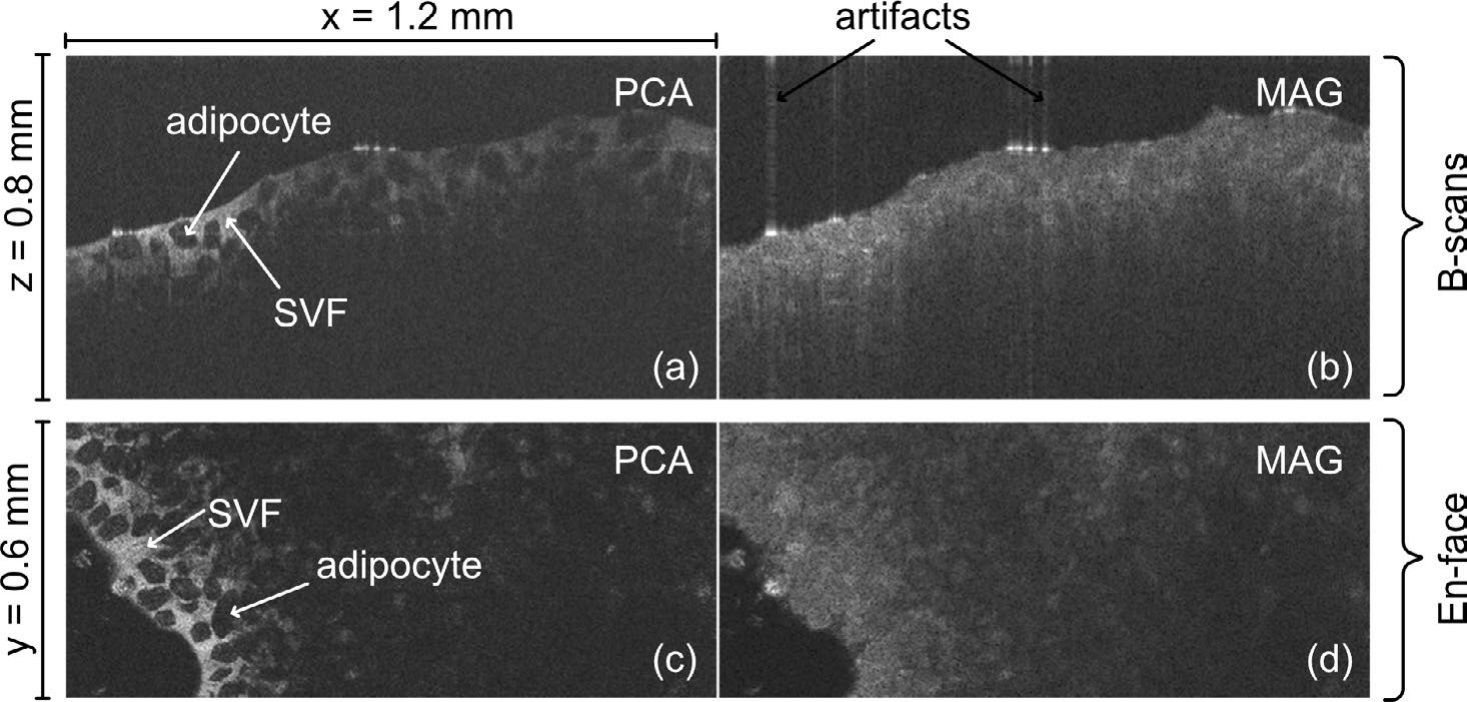
Images of adipose tissue of the kidney. (a, b) are B‐scan images, whereas (c, d) are en‐face images. The images presented in (a, c) were created by averaging the 3rd through the 7th principal components to eliminate the artifacts. Images (b, d) are magnitude images. SVF, stromal vascular fraction.

Artifacts caused by specular reflection are common when using the Gabor fusion technique to improve lateral resolution at depth. An example is shown in Figure 8, which presents B‐scan and en face images of bovine kidney adipose tissue.

Artifacts caused by specular reflections are evident at different focusing depths, as shown on the left side of the magnitude B‐scan image (Figure 8b). When averaging images generated from the 3rd through the 7th principal components, these artifacts are fully removed. Figure 8c,d display *en‐face* images from the same volume as the B‐scans in Figure 8a,b. The artifact in the *en‐face* image in Fig. (d) is not visible because the scattering tissue at that depth is quite homogeneous.

By removing high‐order PCA components, which mainly represent noise, and low‐intensity components from certain structures, the PCA images in Figure 8a,c highlight movement. The black structures observed in both B‐scan and *en‐face* images are adipocytes, indicating no intracellular activity, likely due to the long interval between biopsy and imaging. Bright pixels represent the stromal vascular fraction (SVF) and indicate movement of fluids, fibers, and other supporting cells. These images show structures similar to those reported in [37] by using a dynamic FT‐based MHz‐OCT instrument.

**FIGURE 9:**
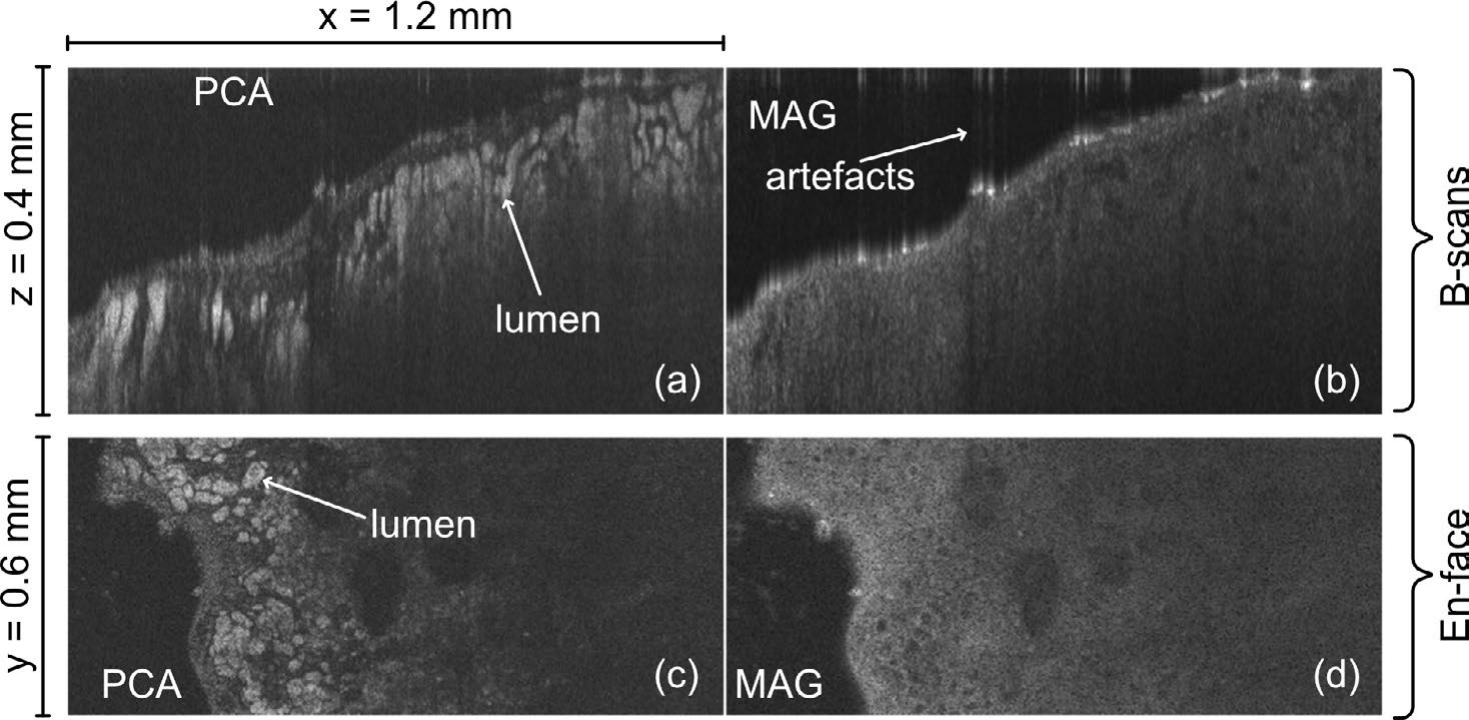
Images of kidney tissue. (a, b) are B‐scan images obtained by Gabor fusing volumes created for five focusing positions, whereas (c, d) are en‐face images obtained for a particular position of the focus. The images presented in (a, c) were created by averaging the 5th through the 7th principal components to eliminate the artifacts for each of the focusing positions. Images (b, d) are magnitude images.

A further example is shown in Figure 9, which shows Gabor‐fused images of bovine kidney tissue. The top images show B‐scans obtained using the PCA approach (left) and the magnitude image (right). Artifacts caused by specular reflection are clearly visible across the entire lateral extent of the magnitude image and appear much reduced on the PCA image. By selecting the principal components more appropriately when constructing the images, these artifacts can be entirely eliminated; however, this can affect the quality of the movement image. To create the image shown in Figure 9a, a good balance between image quality and artifact reduction was achieved by averaging the 5th through the 7th principal components. The large white blobs visible throughout the B‐scan view and on the right side of the en‐face image are the lumens of the renal tubules, whose contents (water, glucose, urea, waste, etc.) are still in motion. This contrasts with conventional histology images, where the fluid is washed off before the sample is placed under the microscope. Additionally, on both B‐scan and e*n‐face* PCA‐based images, inflammatory cells, likely lymphocytes and plasma cells, are visible without the need for infiltration or staining procedures. Conversely, in the magnitude images, distinguishing between the lumens and the inflammatory regions is completely impossible.

**FIGURE 10:**
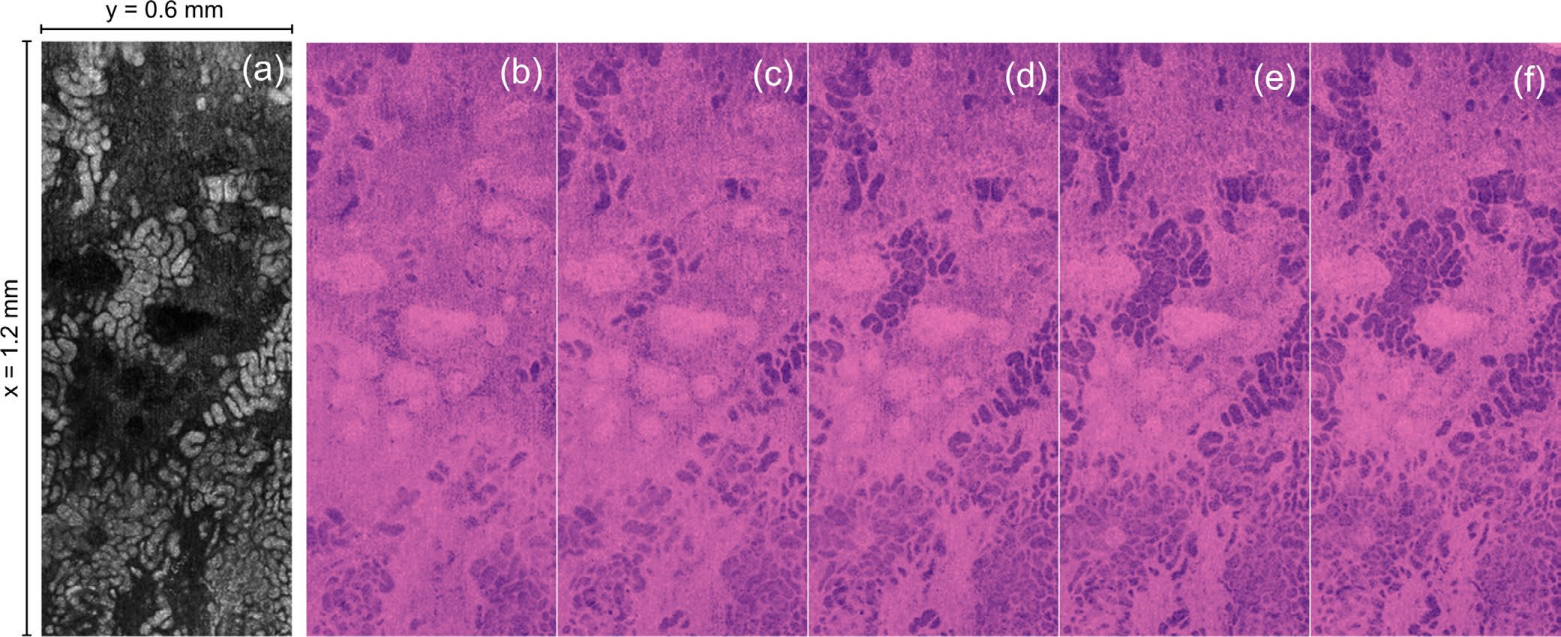
Digital histology *en‐face* images of the kidney tissue obtained after Gabor fusing and flattening. (a) *En‐face* view of a 24 μm‐thick sample, showing lumens across the entire image. (b–f) Color‐inverted histology images of the kidney tissue of thickness 6 μm from various axial positions. The lumens now appear darker.

To produce the fused image shown in Figure 9a, five volumes were used. In each, the focus was positioned just below the sample surface. This allows, after identifying the boundary air/sample using the magnitude images (e.g., Figure 9b), the images to be flattened and histology‐like *en‐face* images of different thicknesses to be produced, similar to those reported in [38]. Examples of these images are shown in Figure 10. In Figure 10a, an *en‐face* image with a thickness of 24 μm clearly shows lumens throughout. Figure 10b–d display *en‐face* views from different axial positions, with a thickness of around 6 μm, similar to those produced by microtomy [39]. To create a pseudo‐histology image, we mapped the dark background to a light background color mode, so the lumens appear darker than in a typical histology image. One must note that these images could be created because the tissue was still hydrated.

## 4 Discussion and Conclusion

This manuscript details the development and use of a phase‐sensitive Dynamic Optical Coherence Tomography device for visualizing motion and cellular activity. The device combines two advanced techniques:

1. Gabor‐domain fusion to address the trade‐off between transverse resolution and depth of focus. By employing a high‐numerical‐aperture objective, a high lateral resolution was achieved, but this limited the native depth of focus to approximately 50 μm. Implementing Gabor‐domain fusion with a variable‐focus liquid lens enables computational merging of datasets from multiple focal planes. This approach extended the focused axial range to 1.9 mm, maintaining signal quality and resolution throughout the volume, and, by using the Master Slave technique to generate images, enabled real‐time OCT image display.
2. Principal Component Analysis. Our experimental results demonstrate that PCA enables effective discrimination between biological motion and static tissue. Specifically, our findings indicate that:
  - The first principal component (PC_1_) can be used to capture static structures such as tube walls and glass plates, whereas higher‐order components (PC_2_‐PC_10_) can effectively isolate dynamic signals, such as Brownian motion and cellular transport.
  - PCA also mitigates saturation artifacts caused by specular reflections, which are common with high‐NA objectives.
  - The system generates “histology‐like” images of bovine kidney tissue, clearly resolving adipocytes, renal tubules, and inflammatory cells. Notably, this was accomplished without the complex infiltration and staining procedures required in traditional histology.

While the proposed method improves DOCT imaging, several technical limitations and challenges still need to be addressed:

- Choosing principal components for the final image is inherently subjective, requiring a careful balance between removing artifacts and preserving motion signals.
- Although PCA effectively separates signals, some artifacts tend to dominate specific components. For example, in lung cell experiments, Fizeau fringes (interference patterns) appeared in PC_2_ and PC_3_, while vertical lines from specular reflections were prominent in PC_4_. This requires a manual exclusion process to produce clean images.
- A specific frequency range cannot be directly linked to a single principal component or a combination of components. However, if needed, a color‐coded mapping of image pixels to specific velocities can be achieved using techniques such as temporal phase shift calculation [12].

Due to the inherent subjectivity in PC selection, future research should focus on developing automated algorithms to identify and select optimal components that maximize the signal‐to‐noise ratio and minimize artifacts. For example, speckle reduction methods utilizing total variation (TV) regularization [40] can be reformulated as constrained PC selection problems. The optimal subset of components may be determined by minimizing a cost function that balances reconstruction fidelity with a speckle penalty. Complex‐correlation‐based approaches [41] also provide an alternative by employing inter‐frame phase coherence as a proxy for the signal‐to‐noise criterion. Finally, self‐supervised methods are particularly well‐suited to this context, as they do not require clean ground‐truth images. Architectures designed for 4D‐OCT volumes [42] or the speckle‐splitting strategy of SSN2V [43] could be adapted to learn optimal principal component subspace selection directly from data statistics, eliminating the need for manual intervention.

Once the appropriate PC components are identified, they can be applied to similar samples under identical experimental conditions. To verify that the PC selection can be reused, we simply need to examine the variance of each component. PCA performs a change of basis in the signal space, projecting the data onto an orthogonal set of principal axes. Unlike the FFT‐based method conventionally employed in DOCT, where each basis function corresponds to a single frequency, each PC generally carries a mixture of spectral contributions. As a result, the ratio $\lambda_{i}/\sum \lambda_{j}$ (with *λ*_*i*_ the i‐th eigenvalue of the covariance matrix) provides a natural, objective metric for ranking components, making the selection criterion reproducible across measurements of the same sample type. Color‐based visualization follows a similar approach: just as the FFT method assigns a color to each frequency band, weighted by its spectral energy, each PC is assigned a color weighted by its variance, *λ*_*i*_. This generalizes the spectral color map to a data‐driven basis, in which the band energy is replaced by the variance of the scores along each principal axis.

The current findings are based on imaging excised biopsies and cell cultures. Translating this technique to in vivo imaging will require improved management of bulk tissue motion and potentially faster acquisition speeds to reduce physiological motion.

The conventional FFT‐based methods can isolate bulk tissue motion by selecting relevant frequency bands. When using PCA, the analogous operation is not performed in the frequency domain but in the “variance domain”. If bulk tissue motion is spatially coherent across the sample, that is all pixels undergo approximately the same rigid displacement, then this motion will project strongly onto a single dominant eigenvector ***p***_***1***_, since it represents the largest and most correlated source of variance in the dataset. Under this assumption, bulk motion can be isolated and removed by simply excluding PC_1_ from the reconstruction. However, this assumption may not hold across all in vivo conditions, and sample‐specific adaptations may be required. More broadly, the key advantage of PCA over FFT‐based approaches in this context is its ability to discriminate between different motion patterns within the sample. Rather than separating by frequency, PCA separates by covariance structure: two cells exhibiting distinct motility patterns, for instance, one undergoing directed motion and another undergoing Brownian‐like fluctuations, will contribute to different principal components, since their dynamics are statistically decorrelated. Each PC therefore captures a distinct dynamical mode, regardless of whether those modes overlap spectrally.

While the direct correspondence between PCA components and specific time scales is less transparent than in FFT, it is not absent. The power spectrum of each eigenvector ***p***_***i***_, encodes the characteristic frequencies associated with that dynamical mode. This link between PCA and time scale could, in principle, be exploited to partially recover a frequency‐resolved interpretation of the components, representing a promising direction for future work.

The current system utilizes 16 B‐scan repetitions. Future optimization should investigate the extent of data reduction achievable without undermining the statistical validity of the principal component analysis (PCA).

## Author Contributions

Both authors were involved in conceptualization, investigation, data acquisition, post‐processing software development, writing the original draft, and subsequent versions of the document. A.B. developed the experimental setup and the data acquisition software.

## Funding

This work was supported by Academy of Medical Sciences (SBF007\100162), Ministerio de Ciencia Innovacion y Universidades (BG24/00089), Royal Sociey (RGS/R1/221324).

## Conflicts of Interest

Adrian Bradu is a co‐inventor of patents for the University of Kent.

## Supporting information


**Figure S1:** B‐scan images of a plastic tube filled with milk corresponding to the first 15 principal components.
**Figure S2:** SNR (a) and CNR (b) values calculated for a number of 32 752 combinations of principal components.
**Figure S3:** B‐scan images of a bovine kidney tissue reconstructed using various sets of principal components as shown in the callouts.

## Data Availability

Data underlying the results presented in this paper are not publicly available at this time but may be obtained from the authors upon reasonable request.
