# Supplementary material for: Using Principal Components Analysis to Visualize Motion and Mitigate Artifacts in Dynamic Optical Coherence Tomography
Source: J Biophotonics. 2026 Jun 23;19(6):e70315. doi: 10.1002/jbio.70315 (PMC13291352; doi:10.1002/jbio.70315)
Supplement: Supplementary file 1 — Figure S1: B‐scan images of a plastic tube filled with milk corresponding to the first 15 principal components. Figure S2: SNR (a) and CNR (b) values calculated for a number of 32 752 combinations of principal components. Figure S3: B‐scan images of a bovine kidney tissue reconstructed using various sets of principal components as shown in the callouts. [file JBIO-19-e70315-s001.docx]

RESEARCH ARTICLE – Supplementary material

Using principal components analysis to visualize motion and mitigate artifacts in dynamic optical coherence tomography

Alejandro Martínez Jiménez^1,2,3^ | Adrian Bradu^1*^

PCA images of the plastic tube


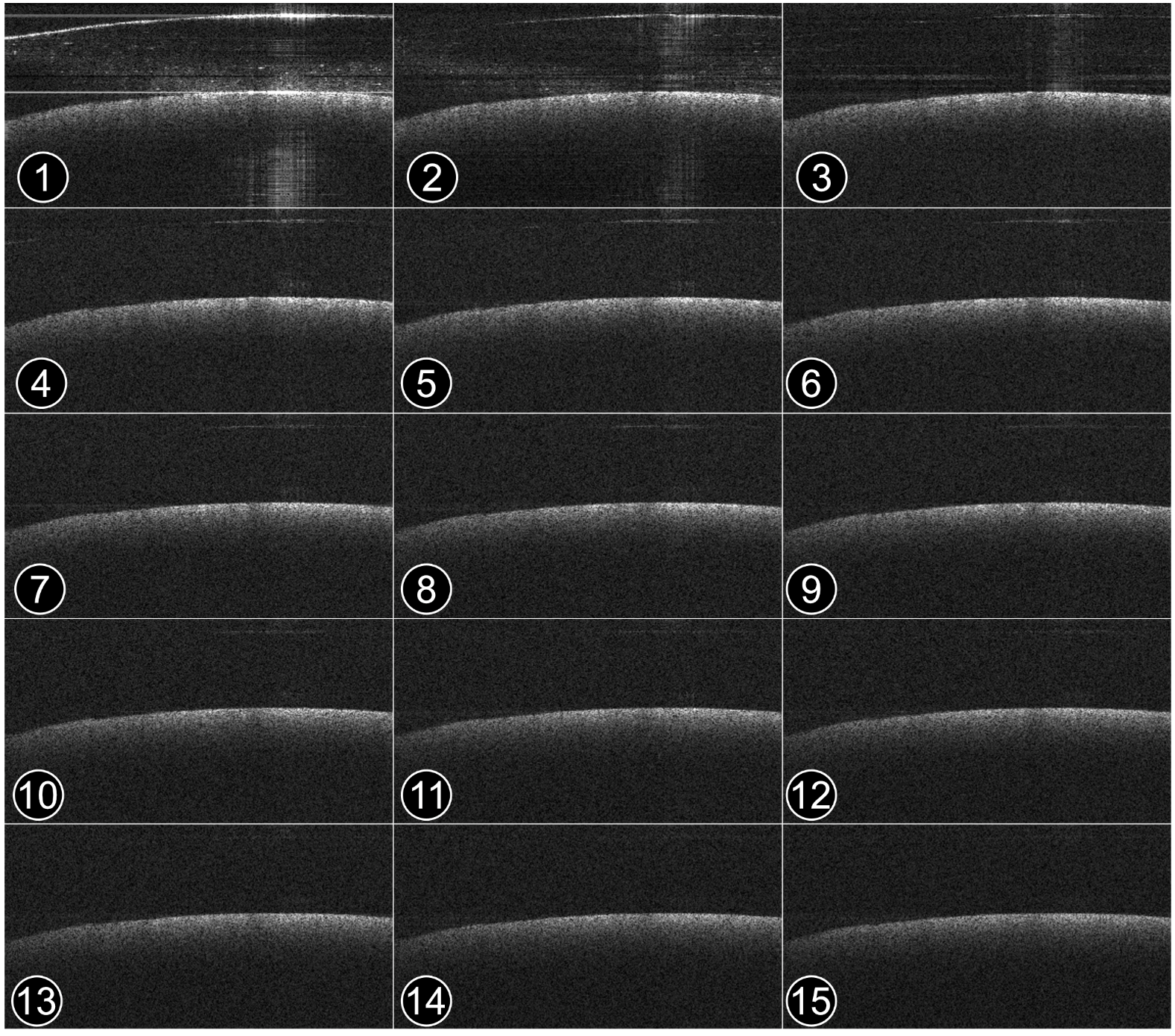


**FIGURE 1S** ǀ B-scan images of a plastic tube filled with milk corresponding to the first 15 principal components.

SNR and CNR calculations when imaging the kidney tissue

32,752 sets of images were obtained using various combinations of the 15 available principal components, and SNR and CNR values were calculated. Typical SNR and CNR values are plotted in the figure below, based on data acquired by imaging bovine kidney tissue. The regions of interest from which these values were obtained are indicated with red circles in Fig. S3. The horizontal axis shows the index associated with a specific set of PCs. Lower values of this index indicate that a small number of PCs were averaged. Some callouts are shown on the graph to indicate exactly which PCs were used to generate the combined image.


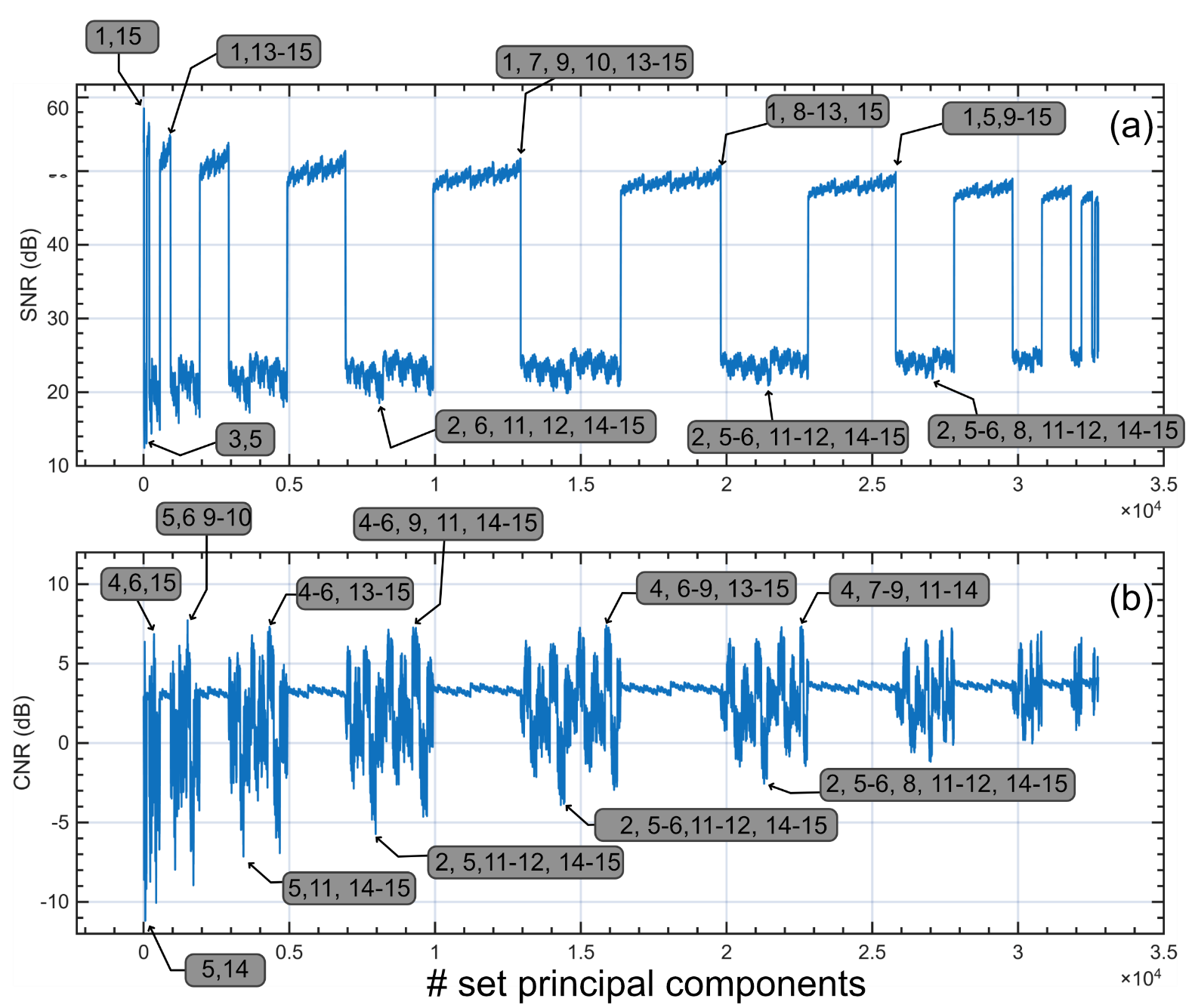


**FIGURE 2S** ǀ SNR (a) and CNR (b) values calculated for a number of 32,752 combinations of principal components.

It is evident that the largest SNR values are always obtained when PC_1_ is employed, and the smallest when it is not. The largest CNR values are obtained when the SNR is small, and the contribution of the static tissue is suppressed, indicating that the set of PCs should be selected to maximize CNR. This is supported by the images in Fig. 3S, which show 6 B-scans of kidney tissue. When using only PC_1_ to generate the B-scan, structures such as the lumens are not distinguishable in the image, whereas artifacts such as horizontal lines are clearly visible (Fig. 3S(a)). The SNR calculated for the red-encircled area exceeds 55 dB. By adding the principal components 7, 9-10, and 13-15 to the reconstruction, Fig. 3S(b) is produced. The SNR is still over 50 dB, and the CNR is about 3 dB. Features responsible for movement begin to appear in the image only when PC_1_ is not employed (Fig. 3S(c)). For this situation, however, the CNR is only about -3.5 dB. By further dropping the low-order PCs, the structures within the tissue are revealed clearly, as illustrated in Fig. 3S(b, d, f), with the best image in Fig. 3S(f), where the CNR is about 8 dB.


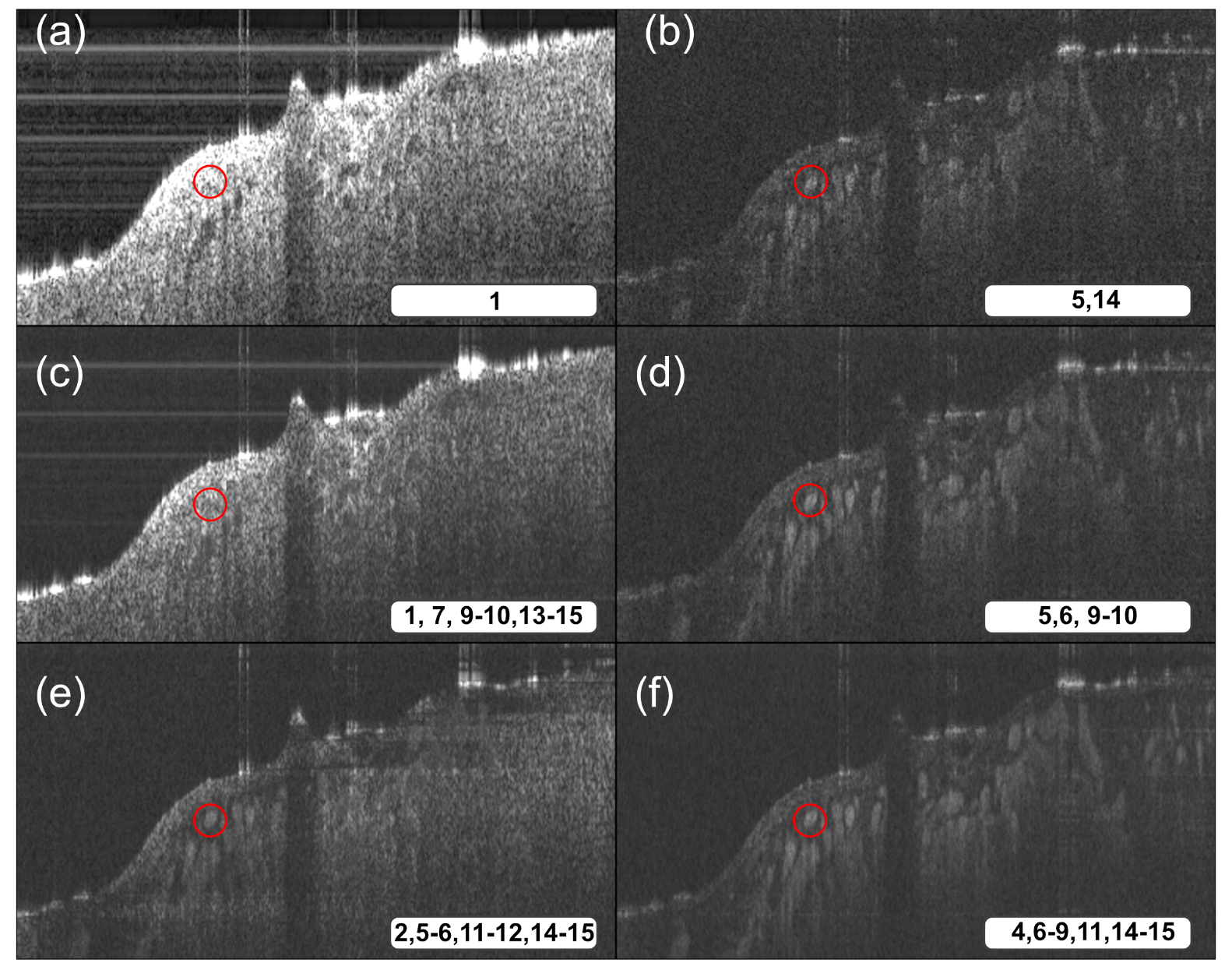


**FIGURE 3S** ǀ B-scan images of a bovine kidney tissue reconstructed using various sets of principal components as shown in the callouts.
